# A Sensor-Aware Physics-Based Framework for Continuous Smartphone Battery State and Lifetime Prediction

**DOI:** 10.3390/s26154707

**Published:** 2026-07-24

**Authors:** Zihan Wu, Wenxuan Dong, Ziyan Yang, Mingguang Diao

**Affiliations:** School of Artificial Intelligence, China University of Geosciences (Beijing), Beijing 100083, China; 1004236204@email.cugb.edu.cn (Z.W.); 1004236116@email.cugb.edu.cn (W.D.); 1004236203@email.cugb.edu.cn (Z.Y.)

**Keywords:** battery state perception, smartphone sensing, state-of-charge prediction, time-to-empty estimation, physics-informed modeling, equivalent circuit model

## Abstract

This paper proposes a continuous-time physics-based framework for smartphone battery state-of-charge (SOC) and time-to-empty (TTE) prediction using multi-source sensing and device operating information. A first-order RC equivalent circuit representation is introduced and extended by incorporating six major factors, including CPU workload, GPS activity, screen brightness, network connectivity, temperature, and battery aging, to characterize realistic battery depletion behavior. Based on the GreenHub dataset, a multi-factor coupled SOC model is developed, and K-means clustering combined with the fourth-order Runge–Kutta algorithm is employed to predict TTE under representative smartphone usage scenarios. Furthermore, an interpretable factor analysis framework integrating sensitivity coefficients, partial contribution rates, and entropy weights is introduced to quantify the contributions of different factors to battery consumption. Experimental results demonstrate that the proposed framework achieves an MAE of 4.2% and an RMSE of 6.8% for SOC prediction. The analysis reveals that CPU workload is the dominant factor affecting battery depletion, while GPS activity and screen usage also exhibit significant impacts. The proposed framework provides an interpretable solution for battery state perception and energy management in intelligent mobile sensing systems.

## 1. Introduction

### 1.1. Background

Smartphones, as indispensable tools for modern communication, work and daily life, have growing energy demands due to expanded functionality. Despite battery technology advancements, their battery life exhibits unpredictable variability, which cannot be solely explained by heavy use but arises from multi-factor interaction. Power consumption is determined by screen parameters, processor load, network and background activity; ambient temperature and the battery’s charge or usage history also significantly affect its effective battery capacity and performance.

Most smartphones are powered by lithium-ion batteries, whose performance is governed by temperature, internal resistance, charge-discharge history and aging. Meanwhile, user behavior such as video streaming, gaming, navigation, and data transmission can result in varying degrees of dynamic power consumption, consuming lithium battery power [1]. Smartphone battery depletion thus fundamentally constitutes a continuous-time energy consumption process driven by multiple coupled factors. Developing a physics-based mathematical model for this process is critical to predicting remaining battery life, identifying the dominant drivers of energy loss, and facilitating the design of intelligent energy management strategies for mobile devices.

### 1.2. Related Work

Battery life prediction and energy consumption modeling have attracted increasing attention in mobile computing. Large-scale datasets such as GreenHub [2] and the recently released BatteryLife benchmark [3] provide valuable resources for investigating smartphone battery behavior and facilitating data-driven prediction methods. Meanwhile, physical and equivalent-circuit-based approaches have been widely adopted to characterize lithium-ion battery dynamics and estimate battery states under realistic operating conditions [4,5].

Recent studies have explored cross-condition generalization for battery lifetime prediction via inter-cell deep learning architectures [6]. Standardized benchmarking platforms including BatteryML deliver unified evaluation frameworks dedicated to battery degradation modeling [7]. Furthermore, chemistry-aware battery degradation models further elevate prediction accuracy under practical cycling scenarios by embedding electrochemical constraints into model formulations [8].

Existing studies have further investigated the effects of sensing operations, CPU workload, wireless communication, GPS activity, and application behaviors on smartphone energy consumption [1,9,10,11,12,13,14]. Although these works have improved the understanding of battery depletion mechanisms, most focus on isolated factors or short-term power estimation. Continuous-time SOC and TTE prediction frameworks that jointly consider multiple coupled factors under realistic smartphone usage scenarios remain relatively limited. This gap motivates the development of the proposed physics-based prediction framework.

### 1.3. Contributions

The main contributions of this work are summarized as follows:

(1) A continuous-time physics-based framework for smartphone battery SOC prediction is proposed inspired by a first-order RC equivalent circuit representation. Combining multi-source sensing data and incorporating six major power-consuming factors, including CPU load, GPS activity, screen brightness, network connectivity, temperature, and battery aging, the framework provides an interpretable representation of realistic battery depletion behavior under diverse operating conditions.
(2) A scenario-driven TTE prediction approach is developed by integrating K-means clustering with the fourth-order Runge–Kutta numerical method. Representative smartphone usage scenarios extracted from the GreenHub dataset are utilized to model nonlinear battery depletion processes and estimate remaining battery lifetime under realistic usage patterns.
(3) An interpretable battery depletion analysis framework is established by combining sensitivity coefficients, partial contribution rates, and entropy weights. Comprehensive validation, sensitivity analysis, and Monte Carlo simulation are further conducted to evaluate model accuracy, robustness, and uncertainty, providing quantitative insights into the dominant factors affecting battery consumption.

**Figure 1:**
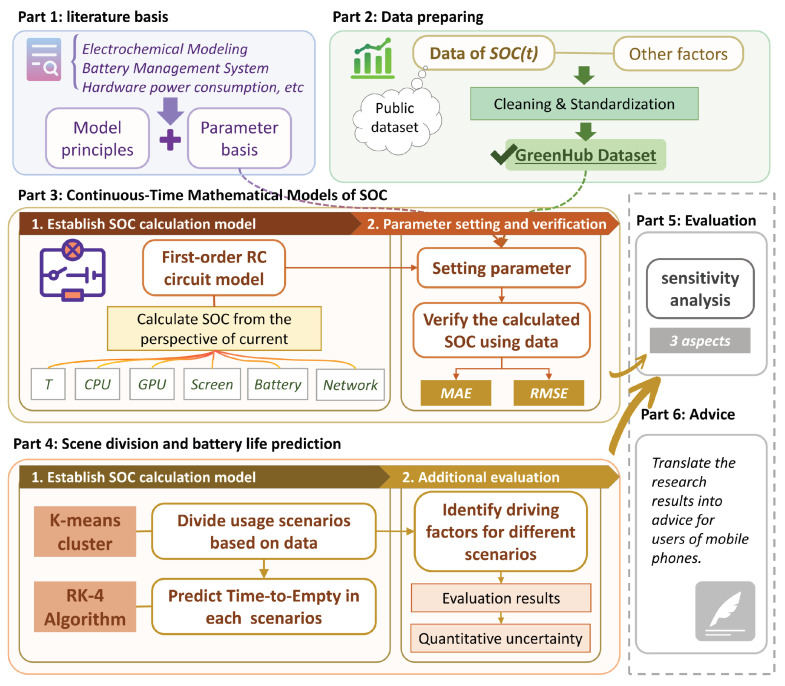
Architecture of the proposed continuous-time SOC and TTE prediction framework.

The overall architecture of the proposed framework is shown in Figure 1. Starting from data preprocessing, the framework integrates continuous-time SOC modeling, scenario-based TTE prediction, and battery depletion factor analysis to provide a comprehensive evaluation of smartphone battery behavior.

## 2. Data Collection and Analysis

This study utilizes the GreenHub dataset [2], a large-scale crowdsourced dataset containing more than 49 million Android smartphone battery and multi-source sensing records. Through data extraction, the dataset fields include 44 core attributes such as battery_level, voltage, temperature, screen_brightness, network_status, and wifi_status, which cover diverse mobile sensing and device operating information. To ensure reliable SOC/TTE prediction and improve model stability, the GreenHub dataset was preprocessed through missing value removal, feature selection, status field encoding, temporal sorting, and numerical standardization. Samples with missing key fields, including battery_level, voltage, temperature, and timestamp, were removed, while redundant attributes weakly related to battery consumption and sensing activities were discarded. Text-based status fields were converted into binary numerical representations, and all records were sorted according to device_id and timestamp to preserve temporal continuity. Finally, Z-score standardization was applied to continuous numerical features to eliminate dimensional differences and improve training stability. During data preprocessing, raw records from the GreenHub dataset are filtered to remove invalid, incomplete, and corrupted samples. Missing values are handled through temporal consistency alignment and exclusion of records with insufficient sensing signals. The proportion of removed samples is relatively small due to the high quality of the dataset. Feature selection is performed based on domain knowledge and relevance to battery state estimation, retaining only sensing variables that have been shown to contribute significantly to SOC and TTE prediction performance.

## 3. Continuous-Time SOC Modeling

### 3.1. Establishment of the Basic Model

To investigate the state of charge (SOC) variation in mobile phone lithium batteries based on mobile sensing data, we constructed a first-order RC analog circuit [4,5]. Due to various sensing and operational factors affecting battery SOC, we selected six main factors for modeling, including screen usage, network connection, GPS connection, CPU usage, battery temperature, and battery aging degree. Based on Kirchhoff’s law and the principles of first-order RC circuits, we perform mathematical modeling transformation on the first-order RC circuit, the circuit state equations shown in Equation (1).

(1) $$\left\{\begin{array}{cc} U_{term}\left(t\right) & =U_{oc}\left(soc\left(t\right)\right)-I_{total}\left(t\right)R_{0}-U_{p}\left(t\right)\\ c_{p}\frac{dU_{p}\left(t\right)}{dt} & =I_{total}\left(t\right)-\frac{U_{p}\left(t\right)}{R_{p}} \end{array}\right.$$

where $U_{term}\left(t\right)$ is the battery terminal voltage, $U_{oc}\left(soc\left(t\right)\right)$ represents the battery electromotive force, $U_{p}\left(t\right)$ is the polarization voltage, $c_{p}$ is the polarization capacitance, $R_{p}$ is the polarization resistance, and $R_{0}$ is the internal resistance of the battery.

In this work, the first-order RC model is introduced to provide a physics-informed representation of battery dynamics and to motivate the derivation of the current-driven continuous-time SOC model.

(2) $$\frac{dsoc(t)}{dt}=-\frac{I_{total}\left(t\right)}{c_{0}}\times 100\%/h$$

where $c_{0}$ is the nominal battery capacity, and the definition formulas of SOC and current is $soc\left(t\right)=\frac{Q(t)}{c_{0}}$ and $i=\frac{dQ(t)}{dt}$.

Because the GreenHub dataset does not provide sufficient electrochemical measurements for online identification of the polarization states and equivalent-circuit parameters, the variables $U_{p}$, $R_{p}$, and $C_{p}$ are not explicitly estimated during model execution.

Consequently, the proposed framework adopts a simplified implementation based on the current balance equation while preserving the physical interpretability inherited from the equivalent-circuit formulation.

The proposed correction terms (temperature effect, aging factor, coupling current, and entropy-weighted contribution) are physics-informed approximations derived from empirical observations and prior literature, aiming to balance physical interpretability and data-driven adaptability in large-scale real-world smartphone scenarios.

### 3.2. Refinement Model Incorporating Influencing Factors

Based on the basic model in Section 3.1, we incorporate factors such as screen size-brightness combinations, processor load, network activity, and temperature, and derive the calculation formula for the total battery current:

(3) $$I_{total}\left(t\right)=I_{screen}\left(t\right)+I_{cpu}\left(t\right)+I_{GPS}\left(t\right)+I_{net}\left(t\right)+I_{couple}\left(t\right)$$

where $I_{total}\left(t\right)$ is the time-varying total battery current, $I_{screen}\left(t\right)$ is the screen current, $I_{cpu}\left(t\right)$ is the CPU current, $I_{GPS}\left(t\right)$ is the GPS current, $I_{net}\left(t\right)$ is the network current, and $I_{couple}\left(t\right)$ is the coupling current.

Screen current is modeled as a function of screen size and brightness:

(4) $$I_{screen}\left(t\right)=I_{screen0}+k_{1}\cdot S\cdot L^{k_{2}}$$

where $I_{screen0}$ is the static screen current, $k_{1}$ is the current coefficient per unit area, *S* is the screen size, *L* is the screen brightness, and $k_{2}$ is the brightness gamma coefficient.

Next, we calculate the CPU current $I_{cpu}\left(t\right)$. Based on CMOS power characteristics, CPU current is modeled as [11]:

(5) $$I_{cpu}\left(t\right)=I_{cpu0}+k_{cpu}\cdot \mu^{\eta }$$

where $k_{cpu}$ is the CPU current coefficient, μ is the load rate, and η is the nonlinear exponent.

GPS current is modeled as a time-varying function of signal acquisition activity [13,14]:

(6) $$I_{GPS}\left(t\right)=e_{1}\cdot F\left(t\right)\cdot \left(1-e^{-e_{2}t}\right)$$

where $e_{1}$ is the GPS current coefficient, and $e_{2}$ is the influence index of signal acquisition, F(t) is probability of GPS activation per unit time.

Network current consumption is modeled by considering both connection maintenance and data transmission:

(7) $$I_{net}\left(t\right)=G\left(t\right)\cdot \left(I_{net0}+k_{net}\cdot v^{m}\right)$$

(8) $$G\left(t\right)=\left\{\begin{array}{cc} 0. & network\;close\\ 1. & network\;open \end{array}\right.$$

where $I_{net0}$ denotes the baseline network current, *v* is the data transmission rate, and *m* is the network-dependent nonlinear coefficient.

To capture the interaction among multiple power-consuming modules, a coupling current term is introduced:

(9) $$I_{couple}\left(t\right)=k_{couple}\cdot \mu \cdot v$$

Moreover, the effective battery capacity $C_{0}$ of the battery is not constant and is significantly affected by ambient temperature. Low temperatures reduce lithium-ion activity, leading to capacity decay, while high temperatures accelerate internal side reactions, also reducing effective battery capacity. Based on the physical properties of lithium polymer batteries and the Arrhenius equation, a temperature nonlinear correction factor is introduced as:

(10) $$k_{temp}\left(t\right)=\frac{2k}{k^{2}+1}$$

(11) $$\ln k=-\frac{E_{a}}{R}\left(\frac{1}{T(t)}-\frac{1}{T_{0}}\right)$$

where $T_{0}$ is 298k, *k* is the relative reaction order of lithium ions, *R* is the molar gas constant, and $E_{a}$ is the activation energy.

Battery aging adversely affects the effective battery capacity of battery capacity. To quantify aging, we use the number of charge-discharge cycles as an indicator and introduce an aging factor $k_{age}\left(t\right)$ for correction. The aging factor is constructed as follows:

(12) $$k_{age}\left(t\right)=e^{-m_{age}\cdot N}$$

This simplified formulation captures only the dominant cycle-aging effect, while more complex degradation mechanisms such as calendar aging, temperature dependence, and state-of-health evolution are not explicitly modeled in this work [15].

Taking into account the refined influence of six factors, finally calibrated capacity and the continuous expression equation of SOC with respect to time are refine to:

(13) $$\left\{\begin{array}{cc} c_{able} & =c_{0}\times k_{temp}\left(t\right)\times k_{age}\left(t\right)\\ \frac{dsoc(t)}{dt} & =-\frac{I_{total}\left(t\right)}{c_{able}}\times 100\%/h \end{array}\right.$$

### 3.3. Model Parameter Determination and Validation

#### 3.3.1. Parameter Estimation

The dataset is split in a temporal and device-aware manner to ensure that data from the same device do not appear simultaneously in both training and testing sets, thereby avoiding data leakage. After filtering invalid records, the remaining dataset is used for model evaluation, and parameter calibration is performed exclusively on the training subset.

The parameter values were determined according to the relative importance reported in previous studies and further adjusted through empirical calibration on the GreenHub dataset. The corresponding literature sources supporting each factor are summarized in Table 1.

Although the proposed framework adopts a single set of calibrated parameters, smartphone hardware heterogeneity inevitably introduces additional uncertainty into battery prediction. The objective of this work is to establish a generalized continuous-time prediction framework applicable to large-scale crowdsourced data rather than to develop device-specific battery calibration models.

Future work will investigate adaptive parameter identification and device-specific calibration strategies to further improve prediction accuracy across different smartphone platforms.

#### 3.3.2. Model Parameters and Structural Rationality Verification

To evaluate the prediction accuracy of the proposed Continuous-Time SOC Model, the GreenHub dataset was used for validation by comparing predicted SOC values with actual battery data. The SOC values were standardized within the range of (0, 100), and the prediction performance was evaluated using MAE, RMSE, maximum positive error, maximum negative error, and error standard deviation.

To further validate the effectiveness of the proposed framework, we introduce several representative baseline methods for comparison, including two widely used deep learning models (LSTM and GRU) and a classical smartphone power estimation method (PowerBooter).

The evaluation results in Table 2 indicate that the proposed model achieves an MAE of 4.2% and an RMSE of 6.8%, demonstrating reliable prediction accuracy and stable computational performance under realistic smartphone usage conditions. Overall, the model exhibits reasonable structural design and satisfactory engineering applicability.

The comparative results demonstrate that data-driven models such as LSTM and GRU can achieve relatively low prediction errors; however, they lack physical interpretability and require significantly higher training complexity.

In contrast, PowerBooter provides improved interpretability but suffers from lower prediction accuracy due to simplified system-level assumptions.

The proposed framework achieves a balanced trade-off between prediction accuracy and physical interpretability, making it more suitable for large-scale real-world deployment.

To ensure a fair and reliable evaluation of the reported results, we further clarify the experimental data partition strategy. The GreenHub dataset is divided using a temporal hold-out approach, where earlier data segments are used for model training and later unseen segments are used for testing. This setting avoids information leakage and better reflects real-world deployment conditions.

In addition, all parameter calibration is performed strictly on the training set, and the testing set is never used during model tuning or coefficient adjustment. Although the dataset contains multi-device and multi-user records, device-specific metadata is not fully available in GreenHub; therefore, the model is evaluated at a population level rather than device-specific stratification. This ensures a consistent and fair evaluation of the proposed framework under heterogeneous smartphone usage conditions.

## 4. TTE Prediction Model

**Figure 2:**
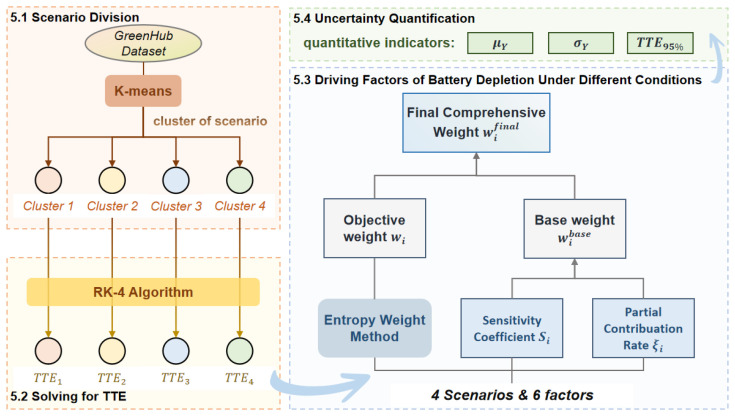
Scenario-driven TTE prediction and factor analysis framework.

To investigate battery depletion under different smartphone usage conditions, a TTE prediction framework was established, as illustrated in Figure 2. The framework mainly includes scenario clustering, TTE estimation, influencing factor analysis, and uncertainty quantification.

### 4.1. Scenario Division

To identify representative smartphone usage patterns, the K-means clustering algorithm is applied to the GreenHub dataset. The clustering process considers six major factors, including temperature, CPU load, GPS status, screen brightness, battery aging level, and network connectivity. All features are normalized before clustering to eliminate scale differences among variables. The number of clusters is determined using the elbow method, and the Euclidean distance metric shown in Equation (14) is used to measure the similarity between samples and cluster centroids.

(14) $${∥x_{i}-c_{j}∥}_{2}=\sqrt{\sum_{d=1}^{r}{\left(x_{id}-c_{jd}\right)}^{2}}$$

where $x_{i}$ is a data point, $c_{j}$ is the centroid of cluster *j*, *r* is the total number of factors, $x_{id}$ is the value of factor *d* for $x_{i}$, and $c_{jd}$ is the value of factor *d* for $c_{j}$.

### 4.2. Solving for TTE

Based on Equation (13), the SOC evolution process can be obtained through numerical integration and subsequently used for TTE prediction. Because the discharge current is nonlinear and varies with operating conditions, the fourth-order Runge–Kutta (RK-4) method is employed to solve the SOC differential equation, as shown in Equation (15).

(15) $$SOC_{n+1}=SOC_{n}+\frac{1}{6}\left(k_{1}+2k_{2}+2k_{3}+k_{4}\right)$$

### 4.3. Analysis of Battery Depletion Drivers Under Different Usage Scenarios

#### 4.3.1. Determining Factor Weights

To quantify the influence of different factors on battery depletion, two complementary indicators are introduced: the sensitivity coefficient and the partial contribution rate. The former reflects the physical impact of a factor on the discharge process, while the latter measures its relative contribution under multi-factor interactions.

(1) Sensitivity Coefficient $S_{i}$

The sensitivity coefficient $S_{i}$ is used to quantify the physical influence of each factor on the battery discharge process and is defined as:

(16) $$S_{i}=\frac{\partial }{\partial x_{i}}\left(\frac{I_{total}\left(t\right)}{c_{able}\left(t\right)}\right)$$

where $x_{i}$ denotes the *i*-th influencing factor (e.g., *L*, α, *T*, $M_{m}$), $I_{total}$ is the nonlinear total discharge current, and $c_{able}$ is the effective battery capacity corrected for temperature and aging effects.

The sensitivity coefficients are normalized as:

(17) $$S_{i}^{norm}=\frac{|S_{i}{\left|-|S\right|}_{min}}{{\left|S\right|}_{max}-{\left|S\right|}_{min}}$$

(2) Partial Contribution Rate $\xi_{i}$

To evaluate the relative importance of each factor under coupled conditions, the partial contribution rate $\xi_{i}$ is introduced. It decomposes the overall TTE variation into independent factor contributions and is calculated as:

(18) $$\xi_{i}=\frac{\Delta v_{i}}{\sum_{j=1}^{n}\left|\Delta v_{j}\right|}$$

where $\Delta v_{i}$ is the contribution of the single factor, and $\sum_{i=1}^{n}\xi_{i}=1$.

The sensitivity coefficient and partial contribution rate are further combined to obtain a base weight for each factor. The resulting scores are normalized to generate comparable factor weights.

#### 4.3.2. Calculating Entropy Weights of Factors

To reduce the subjectivity of the base weights, the entropy weight method is adopted to obtain objective factor weights from the data. Sensitivity coefficients and partial contribution rates are first normalized and organized into an evaluation matrix. Information entropy is then used to quantify the information provided by each indicator, allowing more informative indicators to receive higher weights.

The final comprehensive weight is obtained by combining the physics-based weight with the entropy-based objective weight:

(19) $$\omega_{i}^{final}=\alpha \cdot \omega_{i}^{base}+\left(1-\alpha \right)\cdot \omega_{i}$$

where α=0.5 is selected to assign equal importance to physical mechanisms and data-driven information.

### 4.4. Uncertainty Quantification

To assess prediction uncertainty, Monte Carlo simulation is performed by randomly sampling model parameters within physically reasonable ranges. Repeated simulations generate multiple TTE estimates, from which the statistical characteristics of prediction uncertainty are calculated.

The mean TTE is given by:

(20) $$\mu_{Y}=\sum_{i=1}^{N}TTE_{i}$$

(21) $$\sigma_{Y}=\sqrt{\frac{1}{N-1}\sum_{i=1}^{N}{\left(TTE_{i}-\mu_{Y}\right)}^{2}}$$

(22) $$TTE_{95\%}=\left(\mu_{Y}-1.96\sigma_{Y},\mu_{Y}+1.96\sigma_{Y}\right)$$

where $\mu_{Y}$ denotes the estimated optimal depletion time, $TTE_{i}$ represents the depletion time under the *i*-th parameter group, $\sigma_{Y}$ indicates the estimated standard deviation reflecting the fluctuation extent influenced by parameter uncertainty, and $TTE_{95\%}$ denotes the 95% confidence interval.

## 5. TTE Prediction Results

### 5.1. Scenario Clustering Results

The scenario segmentation, a critical preparatory step for constructing the TTE prediction model, was performed using the k-means clustering algorithm. The clustering results, summarized in Table 3, successfully identified four distinct, representative smartphone usage scenarios, each with unique hardware and application behavior patterns that directly influence battery depletion dynamics.

To further justify the selection of the number of clusters, additional validation metrics are considered beyond the elbow method. Specifically, the silhouette coefficient and Davies–Bouldin index are used to evaluate clustering compactness and separation. These metrics, together with the elbow curve, consistently indicate that four clusters provide a balanced trade-off between intra-cluster similarity and inter-cluster distinction. Therefore, the selection of four usage scenarios is not arbitrary but supported by multiple clustering evaluation criteria.

**Figure 3:**
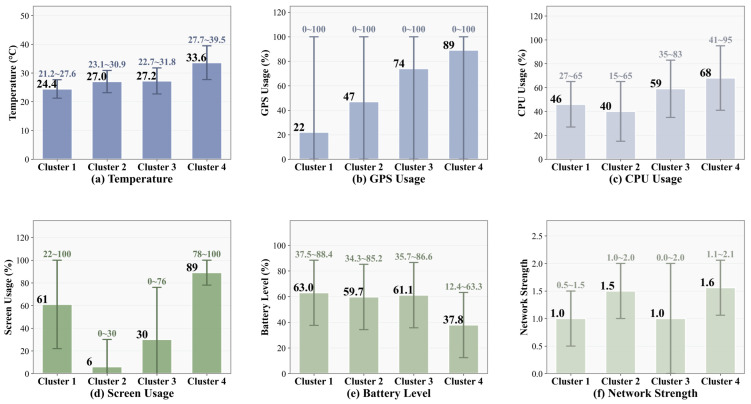
Comparison of six influencing factors across four clustered usage scenarios.

Figure 3a–f present the mean values (bar height) and fluctuation ranges (vertical error bars) of six core factors across the four clustered scenarios.

### 5.2. TTE Under Different Scenarios

This section presents the TTE prediction results under different usage scenarios.

#### 5.2.1. Impact of Initial SOC on TTE

**Figure 4:**
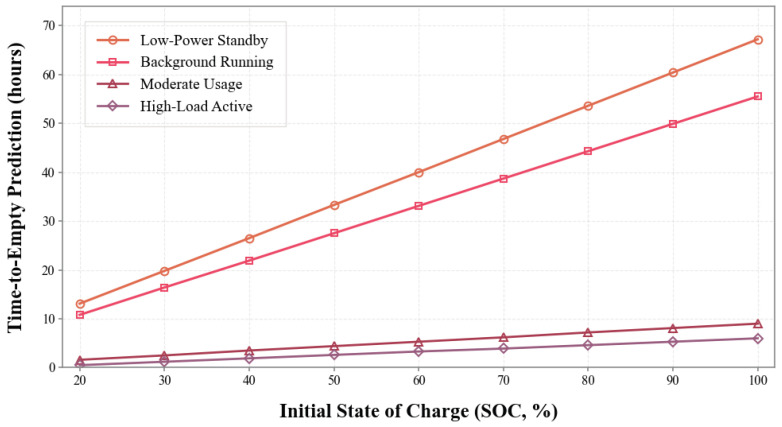
Impact of the initial SOC on TTE under different usage scenarios (results obtained from real data-driven prediction under the proposed framework).

Figure 4 quantifies the relationship between initial SOC and TTE across the four clustered scenarios, providing insights into how starting battery level influences depletion time under different usage patterns.

For all usage scenarios, TTE increases approximately linearly with the initial SOC, indicating that higher initial battery capacity generally leads to longer battery life. However, the influence of initial SOC varies across scenarios. Low-Power Standby shows the strongest TTE response due to minimal power consumption, while High-Load Active exhibits the weakest response because intensive CPU, GPS, and screen activities dominate battery depletion regardless of the initial SOC. Background Running and Moderate Usage scenarios demonstrate intermediate behaviors, where both initial SOC and background or active power consumption jointly affect TTE.

#### 5.2.2. Battery Depletion Processes for Typical User Profiles

**Figure 5:**
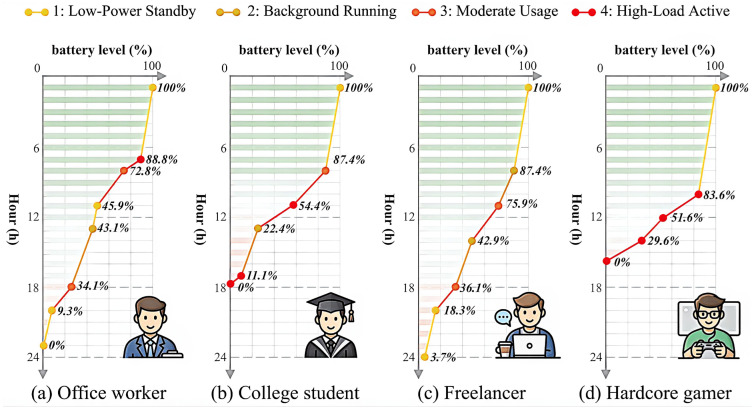
Battery depletion processes for four representative user profiles under scenario-based prediction using real-world smartphone sensing data.

Based on our daily smartphone usage scenario classification, we simulated daily usage for four people (office worker, college student, freelancer, hardcore gamer), with their hourly usage mapped to the four scenarios. Figure 5 presents the computed battery consumption for the groups: vertical axis denotes elapsed time, horizontal axis remaining battery level, and colored lines indicate usage scenarios.

Office workers exhibit the slowest battery depletion, whereas college students and hardcore gamers show significantly shorter battery life due to intensive CPU, GPS, and screen usage. Freelancers demonstrate intermediate behavior with relatively stable battery consumption.

### 5.3. Analysis Results of Influencing Factors Under Different Scenarios

**Figure 6:**
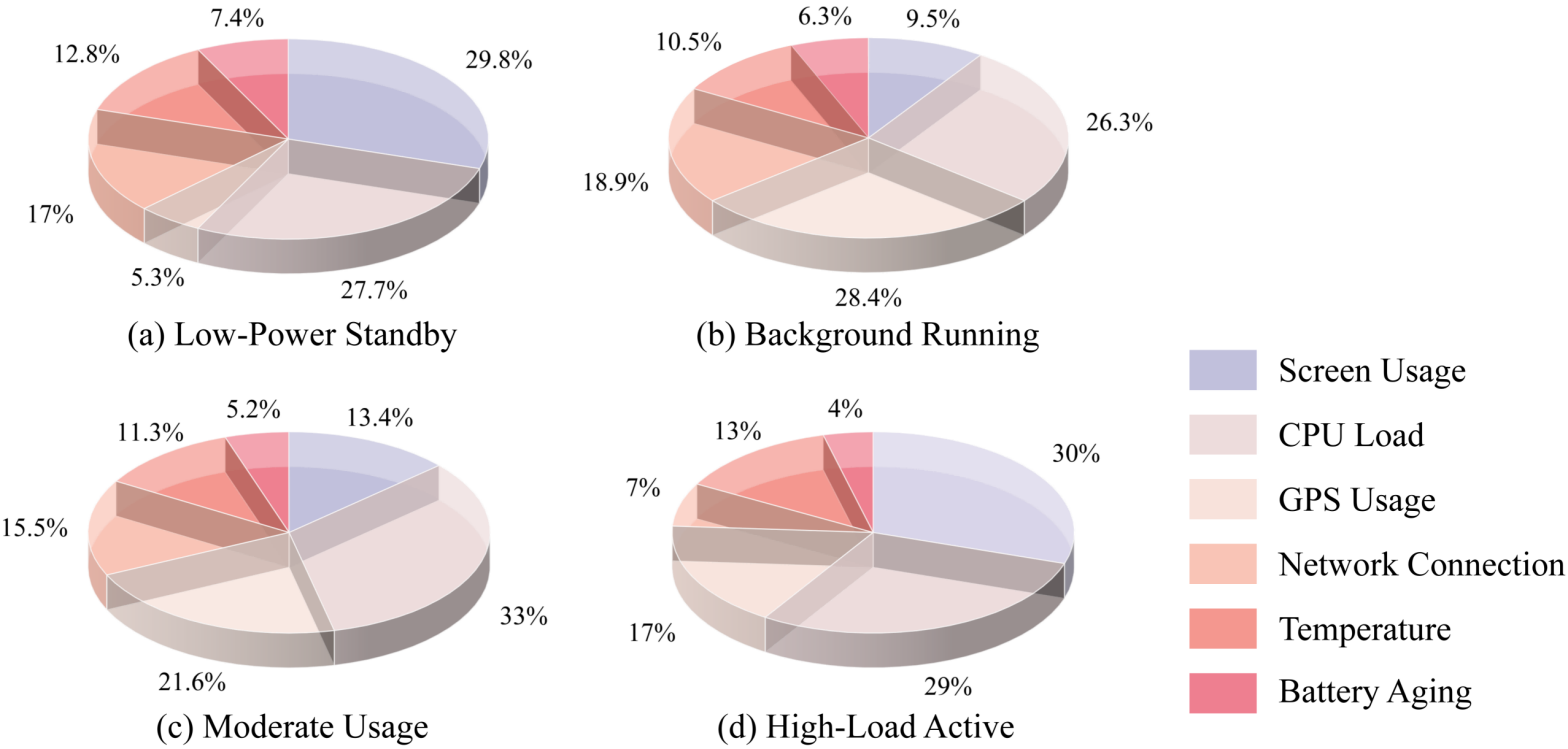
Weights of six influencing factors under different smartphone usage scenarios derived from the proposed scenario-aware prediction framework using real-world data. Note: Minor rounding errors lead to the total percentage being slightly less than 100%.

Based on the coupling weight model of integrated sensitivity coefficient, partial contribution rate, and entropy weight method established in Section 5.2, this section quantifies the weights of six influencing factors in four usage scenarios, as shown in Figure 6.

Figure 6 shows that the dominant factors vary across usage scenarios. CPU load consistently exhibits the highest contribution, particularly under Moderate Usage and High-Load Active conditions. GPS activity becomes more influential in Background Running scenarios, whereas screen usage contributes more significantly in Low-Power Standby. In contrast, battery aging and temperature generally have relatively limited short-term effects.

The results provide calibration support for TTE prediction under different usage scenarios. Among all factors, CPU load consistently exhibits the highest impact on battery depletion, while GPS, network, and screen usage also contribute significantly in certain scenarios due to continuous data exchange and location services. In contrast, battery aging and temperature show relatively low short-term influence on TTE, as their effects mainly accumulate over long-term usage rather than single discharge cycles.

## 6. Sensitivity Analysis

To evaluate the robustness of the proposed TTE prediction framework under realistic smartphone usage conditions, the Moderate Usage scenario was selected as the baseline for sensitivity analysis. The analysis focuses on model assumptions, parameter sensitivity, and uncertainty quantification.

### 6.1. Model Assumption Analysis

Two important assumptions were tested to evaluate their influence on prediction accuracy. First, the coupling current term was removed from the total current equation. Under this condition, the MAE increased from 4.2% to 5.9%, while the RMSE increased from 6.8% to 8.6%, indicating that the coupling effect between hardware modules plays an important role in accurately modeling battery depletion.

Second, the battery aging correction term was removed from the effective battery capacity equation. In this case, the MAE rose to 5.2% and the RMSE increased to 7.7%. Although the impact is smaller than that of the coupling term, the results show that battery aging gradually affects TTE prediction as the charge-discharge cycle count accumulates. These results demonstrate that both assumptions are necessary for maintaining model reliability.

### 6.2. Sensitivity Analysis of Model Parameters

**Figure 7:**
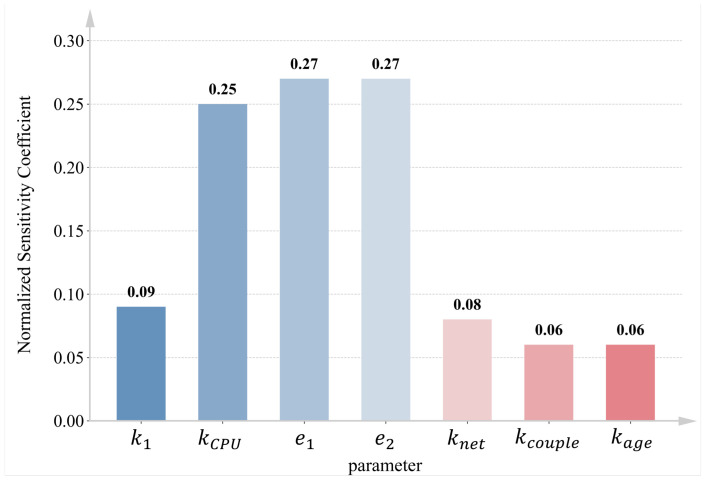
Normalized sensitivity coefficients of model parameters.

Figure 7 shows that the CPU current coefficient has the highest sensitivity among all model parameters, indicating that CPU load fluctuations have the strongest influence on TTE prediction. GPS-related parameters and screen usage exhibit moderate sensitivity, while network, coupling, and aging factors have relatively limited effects. These results demonstrate that CPU activity is the primary driver of smartphone battery depletion, especially under moderate and high-load usage conditions.

### 6.3. Sensitivity Analysis of Model Usage Patterns

**Figure 8:**
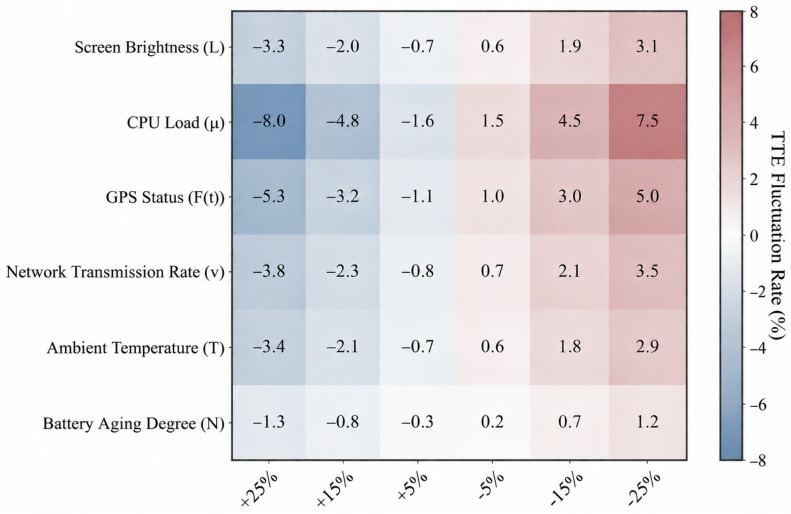
TTE fluctuation rates under different parameter perturbation amplitudes in the moderate usage scenario.

Figure 8 illustrates the influence of parameter fluctuations on TTE prediction under different usage patterns. The results show that CPU load has the greatest impact on TTE, with ±25% fluctuations producing the largest prediction changes, while GPS usage exhibits the second-highest sensitivity. In contrast, battery aging has relatively limited short-term influence. The model also demonstrates stable nonlinear behavior under both positive and negative parameter perturbations.

### 6.4. Sensitivity Analysis Results

To ensure reproducibility of the uncertainty analysis, the Monte Carlo simulation is implemented with the following settings. Key model parameters are assumed to follow normal distributions centered at their calibrated values, with standard deviations determined from empirical variations in the GreenHub dataset. A total of 10,000 Monte Carlo trials are conducted for each usage scenario to ensure statistical convergence. Parameter sampling is performed independently across different model factors, and no cross-correlation is assumed due to the absence of joint distribution information in the dataset.

Monte Carlo simulation was employed to quantify prediction uncertainty under four representative usage scenarios. The relative standard deviations are all below 10%, demonstrating that the proposed framework maintains low-to-moderate uncertainty and satisfies engineering reliability requirements [4]. The detailed statistical outcomes of Monte Carlo simulation for diverse smartphone usage scenarios are summarized in Table 4.

The confidence intervals further indicate that the model achieves stable predictive performance across different usage conditions. In particular, high-load scenarios exhibit lower uncertainty, suggesting that the model performs more consistently under intensive usage patterns. The results confirm the robustness and practical applicability of the proposed TTE prediction framework.

The proposed framework is computationally lightweight, as it mainly relies on algebraic operations and numerical integration (RK4) for continuous-time state estimation. The computational complexity is approximately linear with respect to the number of time steps, O(T). Since no deep neural network inference is required during deployment, the memory footprint and runtime overhead are significantly reduced, making the model suitable for resource-constrained smartphone environments.

## 7. Conclusions

This study proposed a physics-based continuous-time framework for smartphone state-of-charge (SOC) and time-to-empty (TTE) prediction under realistic usage conditions. A continuous-time framework inspired by a first-order RC equivalent circuit representation incorporating six major power-consuming factors, including CPU load, GPS activity, screen brightness, network connectivity, temperature, and battery aging, was developed to characterize battery discharge behavior. Furthermore, K-means clustering and the fourth-order Runge–Kutta algorithm were employed to analyze battery lifetime under representative smartphone usage scenarios.

Experimental results based on the GreenHub dataset demonstrated reliable prediction performance, achieving an MAE of 4.2% and an RMSE of 6.8% for SOC estimation. The proposed framework provides an interpretable approach for continuous-time smartphone battery modeling by integrating physical mechanisms, user behavior characteristics, and data-driven analysis. Compared with purely statistical or machine-learning-based methods, the proposed approach maintains clear physical interpretability while requiring relatively limited training data. The analysis revealed that CPU workload is the dominant contributor to battery depletion, while GPS activity and screen usage also have significant impacts under active operating conditions. Sensitivity analysis and Monte Carlo simulation further confirmed the robustness and stability of the framework under parameter uncertainty and varying usage conditions.

Although the model achieves satisfactory prediction accuracy, the parameters are primarily derived from literature-based calibration rather than device-specific measurements, which may limit its applicability to individual smartphone models. Moreover, the use of population-level parameters may limit the applicability of the framework to individual smartphone models with significantly different hardware characteristics. Future work will explore adaptive online parameter identification and device-specific calibration schemes, with particular focus on adaptive parameter estimation and framework extension to other battery-powered equipment including tablets, wearables, and IoT terminals.

## Figures and Tables

**Table 1:** Calibrated model coefficients and supporting literature sources.

Coefficient Name	Symbol	Value	Core References
The Screen Current Coefficient per Unit Area	$k_{1}$	2.0	[4,16]
The CPU Current Coefficient	$k_{CPU}$	0.045	[5,11]
The GPS Current Coefficient	$e_{1}$	0.05	[12,13]
The Signal Acquisition Impact Index	$e_{2}$	1.0	[13,14]
The Network Current Coefficient	$k_{net}$	0.03	[5]

**Table 2:** SOC prediction performance comparison on the GreenHub dataset.

Method	MAE (%)	RMSE (%)	Max Positive Error (%)	Max Negative Error (%)	Std. Dev. of Error (%)
Coulomb Counting	7.8	10.5	+15.2	−20.6	5.4
PowerBooter	5.6	8.4	+11.3	−14.8	4.2
LSTM	3.9	6.2	+7.5	−10.9	3.0
GRU	4.1	6.5	+7.9	−11.4	3.2
Proposed Method	4.2	6.8	+8.3	−12.1	3.1

**Table 3:** Characteristics of the four clustered usage scenarios.

Cluster	Number of Data	Name	Scene Features
1	426	Low-Power Standby	Low temperature, moderate CPU usage, screen on most of the time, rarely uses GPS.
2	672	Background Running	Moderate temperature, low CPU usage, rarely on screen, frequent GPS use.
3	508	Moderate Usage	Moderate temperature, high CPU usage, occasional screen on, frequent GPS use.
4	394	High-Load Active	High temperature, high CPU usage, screen frequently on, GPS continuously active.

**Table 4:** Monte Carlo simulation results under different usage scenarios.

Name	$\mu_{Y}\left(h\right)$	$\sigma_{Y}\left(h\right)$	${\mathrm{TTE}}_{95\%}$
Low-Power Standby	66.5	3.98	[58.7, 74.3]
Background Running	55.3	2.48	[50.4, 60.2]
Moderate Usage	8.9	0.41	[8.1, 9.7]
High-Load Active	5.9	0.29	[5.3, 6.5]

## Data Availability

The GreenHub dataset used in this study is publicly available through the official project website and Kaggle repository: https://greenhubproject.org/dataset (accessed on 5 March 2026) and https://www.kaggle.com/datasets/hmatalonga/greenhub-farmer (accessed on 5 March 2026). The raw data are publicly accessible from these sources. The processed datasets generated during this study, including the cleaned, filtered, and feature-engineered data used for model training and evaluation, are available from the corresponding author upon reasonable request.
